# Comparative Analysis of B-Cell Receptor Repertoires Induced by Live Yellow Fever Vaccine in Young and Middle-Age Donors

**DOI:** 10.3389/fimmu.2018.02309

**Published:** 2018-10-09

**Authors:** Alexey N. Davydov, Anna S. Obraztsova, Mikhail Y. Lebedin, Maria A. Turchaninova, Dmitriy B. Staroverov, Ekaterina M. Merzlyak, George V. Sharonov, Olga Kladova, Mikhail Shugay, Olga V. Britanova, Dmitriy M. Chudakov

**Affiliations:** ^1^Adaptive Immunity Group, Central European Institute of Technology, Brno, Czechia; ^2^Faculty of Bioengineering and Bioinformatics, Lomonosov Moscow State University, Moscow, Russia; ^3^Center of Life Sciences, Skolkovo Institute of Science and Technology, Moscow, Russia; ^4^Genomics of Adaptive Immunity Department, Shemyakin and Ovchinnikov Institute of Bioorganic Chemistry, Moscow, Russia; ^5^Department of Molecular Technologies, Pirogov Russian National Research Medical University, Moscow, Russia; ^6^Laboratory of Genomics of Antitumor Adaptive Immunity, Privolzhsky Research Medical University, Nizhny Novgorod, Russia

**Keywords:** immunoglobulin repertoire, vaccination, age, yellow fever, plasma cell

## Abstract

Age-related changes can significantly alter the state of adaptive immune system and often lead to attenuated response to novel pathogens and vaccination. In present study we employed 5′RACE UMI-based full length and nearly error-free immunoglobulin profiling to compare plasma cell antibody repertoires in young (19–26 years) and middle-age (45–58 years) individuals vaccinated with a live yellow fever vaccine, modeling a newly encountered pathogen. Our analysis has revealed age-related differences in the responding antibody repertoire ranging from distinct IGH CDR3 repertoire properties to differences in somatic hypermutation intensity and efficiency and antibody lineage tree structure. Overall, our findings suggest that younger individuals respond with a more diverse antibody repertoire and employ a more efficient somatic hypermutation process than elder individuals in response to a newly encountered pathogen.

## Introduction

A number of previously published studies suggests that the function of adaptive immunity is impaired in aged individuals (1, 2). The findings include functionally exhausted immune repertoire displaying a substantially lower diversity of T cell and B cell receptors compared to young individuals (3–6), impaired antigen-driven selection mechanisms (7, 8), and attenuated response to vaccines (9–16).

Functional defects of antibody-mediated vaccine-induced immunity in elderly adults are manifested both in the hampered generation of a primary response and in decreased effect of booster vaccination: low production of vaccine-specific antibodies, low affinity and opsonic capacity of generated antibodies, reduced vaccine longevity (9, 13, 14, 17–23). Altogether, this leads to lower protection achieved in the elderly than in young adults. However, the exact reasons of poor vaccine response in old people have not been fully elucidated.

Recent advances in high-throughput sequencing (HTS) allow performing a targeted readout of hundreds of thousands of B-cell receptor (BCR) heavy chain (IGH) sequences from samples of interest (24–30), providing a powerful tool for investigation of age-related changes in B cell immunity. HTS profiling of BCR repertoires reveals contracted clonal diversity both in naive and antigen-experienced B memory subsets, and accumulation of highly mutated immunoglobulin genes and persistent clonal expansions with aging (25, 31, 32). The latter resembles the age-related changes in T cell repertoire (5, 33–35), and altogether these effects can be linked to the decreased efficiency of vaccination in the elderly adults (32, 34, 36).

The HTS approach was also employed for studies of influenza (25, 27), tetanus (37), and hepatitis B (38, 39) vaccines. It was demonstrated that B cell repertoire has the ability to rapidly expand and contract in a highly dynamic mode in response to vaccination (27). Stereotypic changes of B cell repertoires include increase in mutation frequency and decrease in diversity 4–10 days after vaccination, which corresponds to the maximum concentration of mutated plasma cells released into the peripheral blood (38–40). Interestingly, highly homologous “public” BCR variants can be produced in response to the same antigen in different individuals by convergent recombination and selection (27, 41, 42).

There is also an increasing number of data characterizing changes in the antibody repertoires with respect to vaccine immune stimulus and age. We have found three HTS-based studies of BCR repertoires aimed at revealing the age-related differences in vaccine response, all tracking the changes upon influenza vaccine challenge (25, 32, 43).

Wu et al. (43) analyzed cDNA-based BCR repertoires obtained from the peripheral blood mononuclear cells (PBMC) of young (19–25 years) and old (70–89 years) individuals, where responding B cell clones (groups of homologous clonotypes) could be distinguished by their large size at D7 in terms of the number of included clonotypes. In the old individuals, they reported decreased average clone size within IgA isotype, and increased CDR-H3 length and lower mutation frequency for the large IgA and IgM clones.

Jiang et al. (25) analyzed cDNA-based BCR repertoires obtained from PBMC of 8–17, 18–32, and 70–100 years old groups of individuals at D0, D7-8, and D28 (±4) after vaccination. At D7-8, plasmablasts were sorted as CD3–CD19+CD20–CD27+CD38+ cells. The oldest age group was characterized by fewer B cell lineages compared to other age groups both in PBMC samples obtained before and after vaccination and within the vaccine-responding plasmablast repertoire.

de Bourcy et al. (32) analyzed cDNA-based BCR repertoires obtained from the PBMC of young (21–27 years) and old (73–93 years) individuals, where responding B cell lineages were distinguished as those that were detected at both D0 and D7 and increased their transcript abundance between these time points. They reported reduced intralineage mutational diversification, and decreased proportion of radical (prominently changing the amino acid properties) mutations in the clones responding to vaccination in old individuals. These observations may indicate generally impaired affinity maturation in the old age, as well as accumulated original antigenic sin and the requirement of only fine-tuning of the existing flu-specific memory B cell repertoire in old individuals with long history of response to influenza (32).

All these data have highlighted the importance of B cell repertoire dynamics consideration in vaccine studies in the elderly adults, but were limited to tracking the response to a common pathogen with a substantial exposure history. Here we focused on investigation of the age-related differences in BCR repertoire structure of the plasma B cells responding to the live yellow fever (YF) virus vaccine in young (19–26 years old) versus middle-age (45–58 years old) individuals as a model of response to a previously unencountered pathogen.

We utilized our protocol based on 5′-RACE with unique molecular identifiers (UMI) that allows nearly error-free, full-length (FR1-FR4 plus IgD/IgM/IgG/IgE/IgA isotypes identification) sequencing of IGH variable region repertoires (44), with minor modifications. Given that sufficient coverage is achieved in terms of sequencing reads per cDNA molecule, the use of UMIs dramatically increases the quality of long range high-throughput sequencing, and endues the algorithms of PCR errors correction with high power and precision, critical for resolving the true somatic hypermutation events (44–47).

To focus the analysis on the immunoglobulin repertoires specifically responding to vaccination, we isolated CD20-CD19+CD27^high^CD38^high^ plasma B cells from peripheral blood samples obtained from healthy volunteers 9 days after their first vaccination with live YF vaccine. In this time frame, the concentration of plasma B cells in peripheral blood increases dramatically and mainly represents the cells that respond specifically to the vaccine antigens (40, 48).

It should be noted, that a minor portion of peripheral blood plasma B cells analyzed 9 days after vaccination could include clones responding to the current antigens other than the YF, such as self-antigens and antigens arising from commensal microorganisms or chronic infections. Thus the picture of the differences observed in the plasma B cell repertoires of young and middle-age donors after YF immunization could include imprint from the general differences in the ongoing plasma B cell response between the young and middle-age volunteers, as well as differences in memory-track prehistory of these responses. According to our observations, 9 days after YF vaccination, relative abundance of plasma B cells in peripheral blood increased more than 10-fold and reached 15.5% ± 10% of CD19+CD27^high^ B cells (Supplementary Figure 1), similar for both age groups, which corresponds well to the previous data with influenza vaccination (48). Thus we estimate the contribution of such non-YF vaccination related B cell clones as ~10% of the analyzed cDNA quantity.

It should be also noted that since our approach to immunoglobulin profiling is RNA-based, the resulting IGH repertoires yield the picture that intrinsically accounts for the difference in the IGH mRNA expression levels, thus favoring B cell clones with high production of immunoglobulins.

In our data analysis, we have focused on three groups of variables that together provide a comprehensive repertoire characterization:

1. V-D-J rearrangement structure and CDR3 physicochemical properties;
2. Antibody lineage structure (clonal trees);
3. Profiles of newly acquired and potentially pre-existing somatic hypermutations.

We reveal a number of antibody repertoire features that were distinct between young and middle-age individuals, highlighting age-related differences in humoral immune response directed against newly encountered antigens, that are already detectable by the age of 50.

## Methods

### Blood donors and samples

This study was approved by the local ethical committee and conducted in accordance with the Declaration of Helsinki. All donors were informed of the final use of their blood and signed an informed consent document. The cohort of healthy donors (*n* = 10, Table 1) has been immunized for the first time by the yellow fever vaccine (live freeze-dried preparation of the 17D strain of YFV licensed in Russia, FSUE of Chumakov IPVE, RAMS). Yellow fever is not endemic in Russia and the volunteers have not previously traveled to areas known to be endemic for yellow fever. Peripheral blood (9 ml per sample) was collected on the 9th day after vaccination into EDTA-treated Vacutainer tubes (BD Biosciences). The B cells were stained for surface markers by incubating with following monoclonal antibodies: CD38-PE (clone HB7, eBioscience), CD19-FITC (clone J3-119, Beckman Coulter), CD20-Vio Blue (clone LT20, Miltenyi Biotec), CD27-PC5 (clone O323, eBioscience). The plasma B cells were gated as CD20-CD19+CD27+CD38^high^ and collected directly into RLT buffer (Qiagen) for storage and RNA extraction. The numbers of sorted plasma cells per sample are shown in Table 1.

**Table 1:** Donor and sample metadata.

**Donor ID**	**Age range (median), years**	**Status**	**Number of sorted plasma cells**	**Portion of cDNA used**	**Number of raw paired-end sequencing reads**	**Number of analyzed IGH cDNA molecules**	**Number of unique IGH clonotypes extracted**
P1	19–26 (19)	Young	6,000	1/30	4,410,583	29,052	3,913
P2			6,000	1/30	3,443,963	17,284	3,590
P3			6,000	1/30	4,254,150	10,593	2,159
P4			6,000	1/30	3,737,681	20,665	2,916
P5			6,000	1/30	2,888,569	18,086	2,563
P6	45–58 (55)	Middle-age	6,000	1/30	1,939,674	19,111	2,529
P7			5,000	1/25	3,133,844	42,768	3,520
P8			6,000	1/30	2,731,660	21,879	2,507
P9			3,000	1/15	2,566,472	11,154	1,761
P10			3,000	1/15	1,845,910	20,102	2,427

### RNA extraction, cDNA libraries preparation, and sequencing

UMI-barcoded IGH cDNA libraries for the vaccinated donors were prepared as described previously (44) with minor modifications which allow to introduce Illumina Nextera adapters and indexes during PCR. Briefly, total RNA was extracted from sorted B cells using RNeasy Micro Kit (QIAGEN) and converted to cDNA using 5′ template switch adapter containing UMI. The cDNA was treated with UDG (NEB), and purified using AMPure Beads (Beckman Coulter). A portion of cDNA equivalent to 200 sorted plasma B cells (Table 1) was used for further PCR amplification. Appropriate amount of cDNA used for the library preparation is critical in order to achieve sufficient coverage in terms of sequencing reads per UMI, which is a prerequisite for the efficient error correction (44–46). IGH libraries were amplified using a set of IGHC-specific and 5′ template switch adapter-specific primers introducing indexed Nextera sequencing adapters. Please refer to the Table 2 for the oligonucleotides used. The resulting libraries were analyzed on 2 runs of Illumina MiSeq, paired-end 310+310 nt sequencing. All 10 samples were analyzed within each run, and results of the 2 runs were pooled before further bioinformatic analysis.

**Table 2:** Oligonucleotides used.

**Primer**	**Application**	**Sequence**
**FIRST-STRAND cDNA SYNTHESIS**		
SmartNNNext	5′–template-switch oligo with sequencing illumina adapter U = dU, rG = riboG	AGATGUGTAUAAGAGACAGNNNNUNNNNUNNNNUCTT(rG)_4_
**IGH cDNA synthesis primer mix**		
hIGG_r1	Primer for cDNA synthesis, human IgG heavy-chain mRNA	GAAGTAGTCCTTGACCAGGCA
hIGM_r1	Primer for cDNA synthesis, human IgM heavy-chain mRNA	GTGATGGAGTCGGGAAGGAAG
hIGA_r1	Primer for cDNA synthesis, human IgA heavy-chain mRNA	GCGACGACCACGTTCCCATCT
hIGD_r1	Primer for cDNA synthesis, human IgD heavy-chain mRNA	GGACCACAGGGCTGTTATC
hIGE_r1	Primer for cDNA synthesis, human IgE heavy-chain mRNA	AGTCACGGAGGTGGCATTG
**FIRST PCR AMPLIFICATION**		
Common primer	Step-out primer, anneals on the switch adaptor	AGATGTGTATAAGAGACAG
**IGH reverse primer mix**		
Common-hIGGE_r2	Nested primer with sequencing illumina adaptor, human IgG/IgE heavy-chain cDNA	AGATGTGTATAAGAGACAGARGGGGAAGACSGATG
Common-hIGA_r2	Nested primer with sequencing illumina adaptor, human IgA heavy-chain cDNA	AGATGTGTATAAGAGACAGCAGCGGGAAGACCTTG
Common-hIGM_r2	Nested primer with sequencing illumina adaptor, human IgM heavy-chain cDNA	AGATGTGTATAAGAGACAGAGGGGGAAAAGGGTTG
Common-hIGD_r2	Nested primer with sequencing illumina adaptor, human IgD heavy-chain cDNA	AGATGTGTATAAGAGACAGATATGATGGGGAACAC
**SECOND PCR AMPLIFICATION**		
F-common	Step-out primer with sequencing and P7 illumina adapters	TCGTCGGCAGCGTCAGATGTGTATAAGAGACAG
R- common	Step-out primer with sequencing and P5 illumina adapters	GTCTCGTGGGCTCGGAGATGTGTATAAGAGACAG
**THIRD PCR AMPLIFICATION**		
Fc_i7^a^	Step-out primer with index 1 illumina adapter	CAAGCAGAAGACGGCATACGAGAT[i7]GTCTCGTGGGCTCGG
Fc_i5^b^	Step-out primer with index 2 illumina adapter	AATGATACGGCGACCACCGAGATCTACAC[i5]TCGTCGGCAGCGTC

a, b *Illumina Nextera index adapters (i5 and i7). See illumina Nextera DNA library preparation reference guide and illumina adapters sequences list for more information*.

### Data preprocessing and analysis

UMI extraction and UMI-based consensus assembling was performed using MIGEC software (45), with a 5 reads-per-UMI threshold. Further reads mapping and clonotypes (unique full length IGH nucleotide sequences) assembling was performed using MiXCR as described previously (44) with some changes in MiXCR analysis pipeline (KAligner alignment algorithm was used that allows to detect indels of more than 2 nt). Resulting clonesets deposited at https://figshare.com/articles/Comparative_analysis_of_B-cell_receptor_repertoires_induced_by_live_yellow_fever_vaccine_in_young_and_middle_age_donors/6853961.

All analyses except for depicted in **Figure 5A** were performed using mean values for repertoire features and summary statistics computed for each donor, statistical testing was performed by comparing values for *n* = 5 young and *n* = 5 middle-age donors.

### Antibody lineage analysis

Reconstruction of clonal trees was done using in-house algorithm that takes into account VJ assignment and NDN sequence of IGH and can be briefly described as follows. First, IGH clonotypes are clustered into groups containing sequences with matched V and J segments. Then, a pairwise comparison is performed for each group: if the K-mer (K = 5) composition of NDN regions of two sequences is highly similar the sequences are considered to originate from one ancestor sequence and connected by an edge on the lineage tree.

An edge connects a pair of clonotypes that are likely to come from a pair of cells, one of which is a hypermutated [bears a B-cell receptor with mutation(s)] sub-variant of another. The direction of edge shows which of the clonotypes is a parent one and which is a child one. As we use full-length immunoglobulin sequencing data, one can infer edge direction using parsimony principle: parent clonotype mutations should be a subset of child mutations.

The similarity is computed by summing the information content of each K-mer (that is, the logarithm of its probability to be found in random VDJ rearrangements), thus K-mers containing many non-template bases are scored the most. The similarity score threshold for drawing an edge was selected according a benchmark performed by *in-silico* mixing Raji hypermutating cell line repertoire and PBMC IGH samples. CDR3 hypermutations were obtained using Smith-Waterman alignment for each pair of connected nodes with different CDR3 sequences. Parsimony principle was applied to remove improbable nodes and infer the direction of edges. In order to eliminate duplicate paths and form a tree structure we next removed all incoming edges except the one with lowest number of mutations. To normalize samples for accurate comparison, we extracted 10,000 randomly sampled IGH cDNA molecules from each dataset.

### CDR3 physicochemical property analysis

Averaged CDR3 physicochemical properties of repertoires accounting for the clonotypes size were computed using custom R script. To estimate the energy of the interaction between CDR3 and a random epitope, we used Miyazawa-Jernigan statistical potential (49), that is based on calculating the frequencies of certain amino acids being in close proximity with each other in available structural data, and assuming that these frequencies follow Boltzmann distribution parameterized by corresponding energy values. For each amino acid among the five positions in the middle of CDR3, we computed the average interaction energy with all 20 amino acids. We then summed values across amino acid residues to estimate the energy of the interaction between CDR3 and a random epitope. Other physicochemical properties were analyzed similarly.

### Selection strength

The selection strength was estimated using BASELINe framework (50) which compares the observed frequencies of replacement and silent mutations with the expected ones. BASELINe was applied to a subset of Variable segment sequences (FR1-FR3) that do not contain indels and the closest germline alleles as a reference. For each donor, clonal groups of sequences were collapsed to consensus sequences using SHazaM R package (51) as recommended by BASELINe authors. The probability density functions of the selection strength were compared using built-in statistical test.

## Results

### CDR3 characteristics

Analysis of the averaged CDR3 characteristics was performed for IgA, IgG, and IgM isotypes, weighted by the abundance of each clonotype (i.e., the input of each clonotype was proportional to its frequency within repertoire), and included CDR3 length, added N nucleotides, and physicochemical characteristics for the five amino acid residues located in the middle of CDR3 [having the highest probability to contact with antigen, by analogy with TCRs, (52)]. The latter included averaged statistical potential of CDR3:epitope interactions [the estimated “energy” of interaction between CDR3 and a random epitope (49)], “strength” of interaction [derivative of “energy,” VDJtools (53)], hydrophobicity (Kidera factor 4) (54, 55), and “volume” (VDJtools, values from: http://www.imgt.org/IMGTeducation/Aide-memoire/_UK/aminoacids/IMGTclasses.html) for the young versus middle-age individuals (see Table 3 for the values used for each amino acid property).

**Table 3:** Values used for CDR3 amino acid properties calculation.

**Amino acid**	**Mjenergy**	**Kf4**	**Volume**	**Strength**
A	−2.8455	−0.27	67	0
C	−3.782	−1.05	86	0
D	−2.116	0.81	91	0
E	−2.141	1.17	109	0
F	−5.017	−1.43	135	1
G	−2.499	−0.16	48	0
H	−2.927	0.28	118	0
I	−4.641	−0.77	124	1
K	−1.789	1.7	135	0
L	−5.023	−1.1	124	1
M	−4.1915	−0.73	124	1
N	−2.349	0.81	96	0
P	−2.443	−0.75	90	0
Q	−2.2505	1.1	114	0
R	−2.402	1.87	148	0
S	−2.308	0.42	73	0
T	−2.6145	0.63	93	0
V	−4.093	−0.4	105	1
W	−4.1375	−1.57	163	1
Y	−3.7505	−0.56	141	1

**Figure 1:**
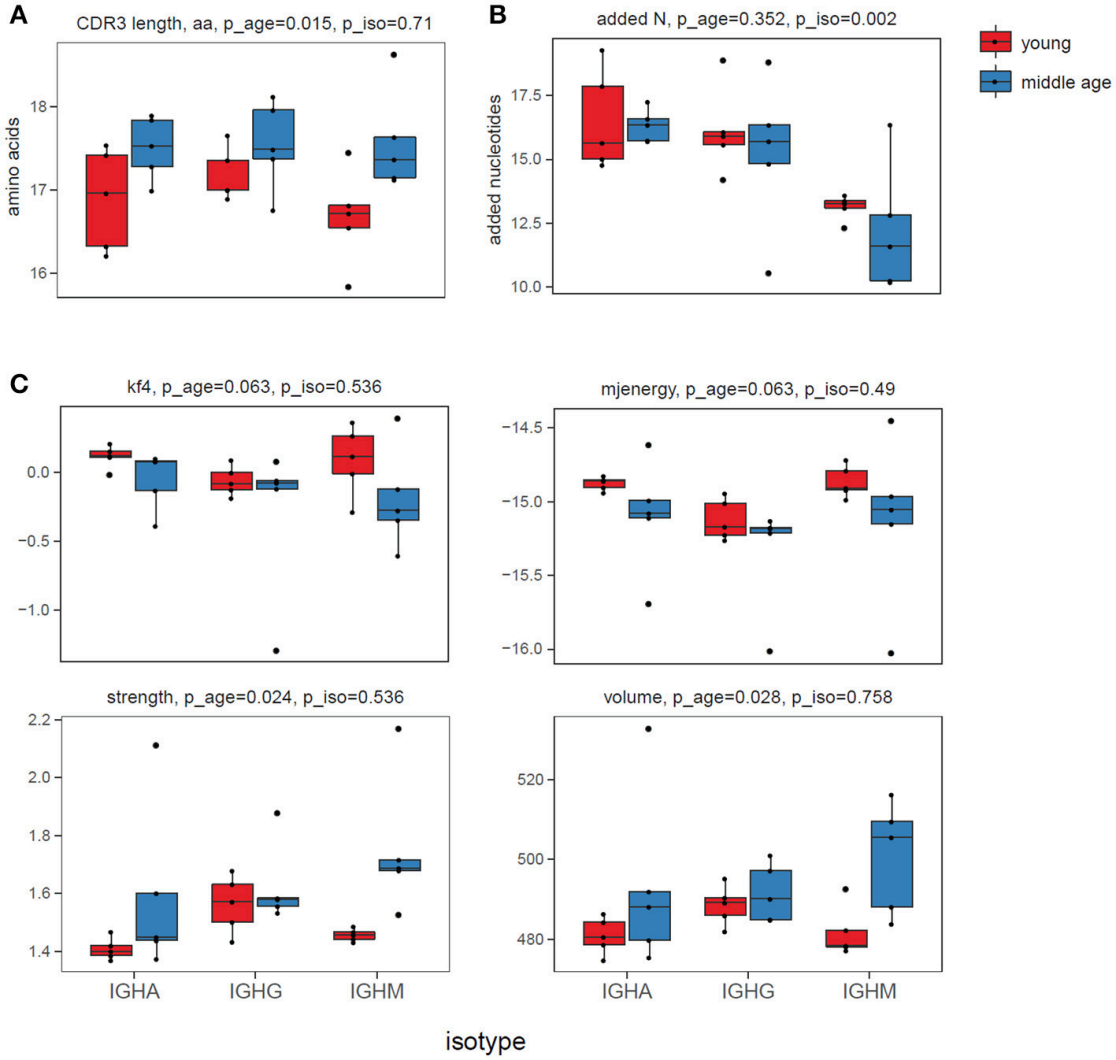
CDR3 characteristics. **(A)** CDR3 length, aa. **(B)** Number of non-template added N nucleotides within V-D-J junction. **(C)** Physicochemical properties for the 5 amino acids residues in the middle of CDR3: Kidera factor 4 (hydrophobicity, lower values refer to more hydrophobic amino acids), potential “energy” of interaction (49) (lower values refer to stronger interaction), “strength” and “volume.” All characteristics were calculated “weighted”—i.e., accounting for IGH clonotype size. ANOVA *p*-values for age and for isotype adjusted using Benjamini & Hochberg correction are shown on top of each plot.

The analysis revealed several features that significantly differed between the responding plasma cell IGH repertoires of the two age groups but not between the isotypes (Figure 1). Of note, differences in “energy,” and hydrophobicity (Kidera factor 4) were previously demonstrated to be critical for antibody affinity and specificity (56). Altogether, observed differences indicated that middle-age individuals tend to respond to a new challenge with IGH variants carrying longer CDR3s [in agreement with (43)], with higher content of bulky, hydrophobic, and strongly interacting amino acid residues in the middle of CDR3.

### Isotype and IGHV gene segments usage

**Figure 2:**
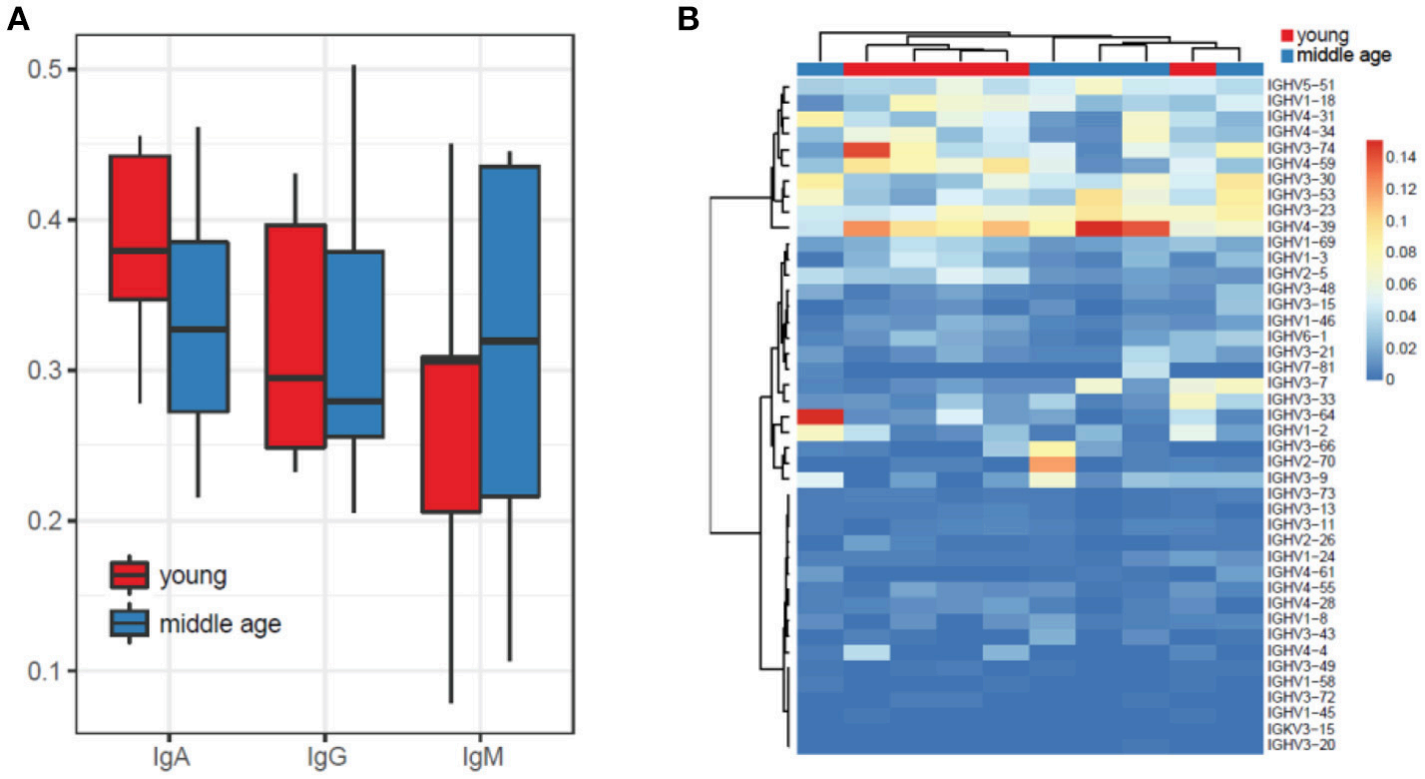
Isotype and IGHV segments usage for YF-vaccinated subjects from the two age groups. **(A)** Isotype usage. **(B)** IGHV usage.

We have not detected prominent differences in IGHV gene segment usage as well as in isotype usage between plasma cell IGH repertoires of young and middle-age individuals vaccinated with YF (Figures 2A,B). The list of most commonly used IGHV segments included the IGHV4 (IGHV4-31, IGHV4-34, IGHV4-59, and IGHV4-39) family, IGHV3 (IGHV3-74, IGHV3-30, IGHV3-53, IGHV3-24) family, as well as IGHV1-18 and IGHV5-51.

Note that potential biases in isotype and IGHV gene segment usage are corrected by UMI-based analysis, since sequencing reads that cover the same cDNA molecule (irrespectively to the efficiency of amplification of each particular isotype or gene segment) are clustered together, each cDNA molecule to a single UMI-labeled group of sequencing reads.

### Differences in immunoglobulin clonal lineage structure

The analysis of antibody repertoires can be extended by grouping IGH clonotypes into clonal lineages (trees) that share a common ancestor (57) and represent a B-cell clone undergoing the affinity maturation process. UMI-based full-length immunoglobulin sequencing (44) and dedicated antibody tree building algorithm (see section Methods) allowed us to accurately infer and analyze clonal lineage structure.

**Figure 3:**
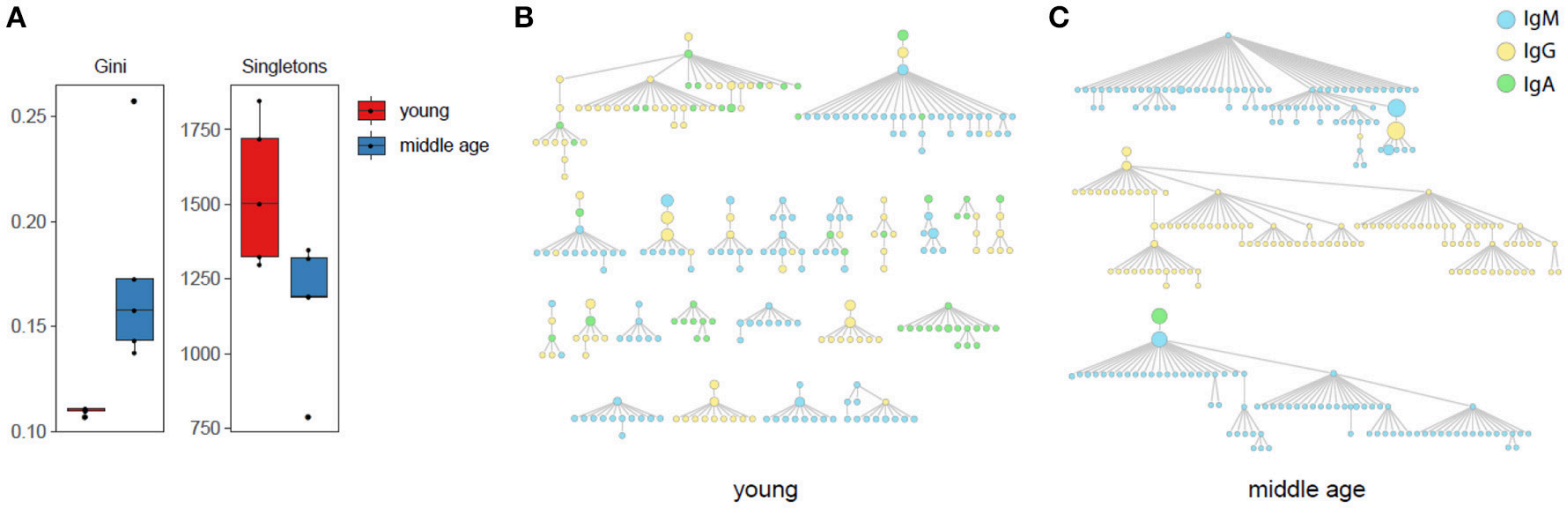
Antibody lineage analysis. **(A)** Diversity of antibody lineages: Gini inequality coefficient for the number of nodes (clonotypes) in a tree (clone), and number of “trees” that include only one node. Both parameters are significantly different (*p* < 0.05) between young and middle-age donors. Two-tailed *T-*test, *p*-values adjusted using Benjamini & Hochberg method. **(B,C)** Top IGH trees by size containing equal number of 270 nodes for united data of all young **(B)** and middle-age **(C)** individuals are shown.

This analysis revealed that basic graph characteristics, such as the Gini inequality coefficient, and number of singletons (clones including only one clonotype) were significantly different between young and middle-age YF-vaccinated donors (Figure 3A). The Gini coefficient measures the inequality of tree size (number of nodes) distribution. Large Gini coefficient values mean that large trees with many mutated variants dominate over small trees, i.e., most of the observed immunoglobulin variants come from few B-cell clones. Smaller values mean that more distinct clones enter the affinity maturation process during an immune response. Thus the direction of the observed differences suggests that young individuals have a more diversified responding repertoire, while middle-age individuals display a more biased lineage architecture with larger trees that account for the majority of observed clonotypes (Figures 3B,C).

Note that the trees may include impossible lineage relations such as IgA to IgM isotype conversion. This reflects the fact that our analysis is limited by sampling depth and particular time point. We do not observe the whole pre-history of hypermutation process, thus additional unseen IGH sequence variants that can resolve this ambiguity may exist, e.g., unseen IgM sequence variant parent to observed IgM and IgA variants.

### Bulk analysis of somatic hypermutations

**Figure 4:**
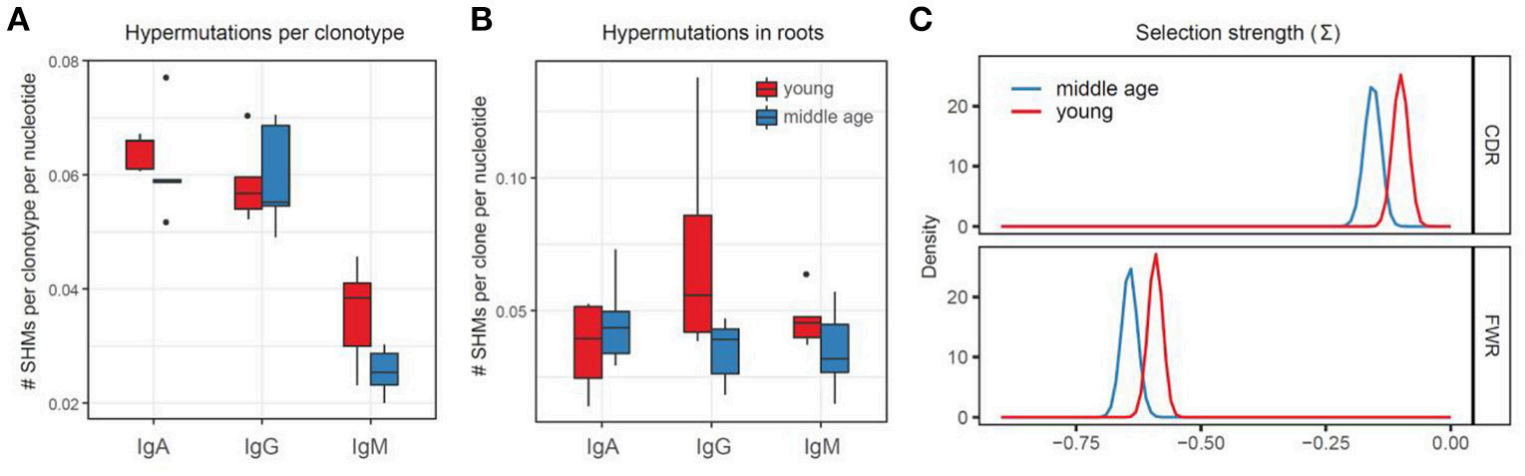
Bulk analysis of somatic hypermutations. **(A)** Somatic hypermutations per clonotype, without using trees information (NS, ANOVA). **(B)** Somatic hypermutations within roots of the trees with >2 nodes (NS, ANOVA). **(C)** Selection strength estimated using BASELINe framework. Adjusted *p* = 0.011 for CDR, 0.011 for FWR.

Bulk load of somatic hypermutations per clonotype obtained without using the information on the trees structure was comparable between young and middle-age plasma cell IGH repertoires (Figure 4A). We controlled for isotype which is necessary in such comparisons as there are substantial differences between base mutation burden for each isotype (e.g., mean number of SHMs in an IgG clonotype is about 2 times higher than per IgM clonotype).

In order to estimate the burden of somatic hypermutations that could earlier accumulate within memory B cells clones currently participating in immune response, we analyzed the frequency of hypermutations within the identified roots of the trees—the IGH sequence variants that were closets to germline within each tree. This analysis also has not revealed significant differences between the young and middle-age individuals in a load of root somatic hypermutations, potentially pre-existing within responding IGH clones (Figure 4B).

To test for the intrinsic differences in the structure of hypermutations, we estimated the average “selection strength” that drived the accumulation of somatic hypermutations in young versus middle-age individual repertoires using BASELINe framework (50), which is based on estimation of expected versus observed frequencies of replacement and silent mutations. This analysis indicated higher “selection strength” in the young versus middle-age individual IGH repertoires (Figure 4C).

### Observed history of ongoing somatic hypermutations

**Figure 5:**
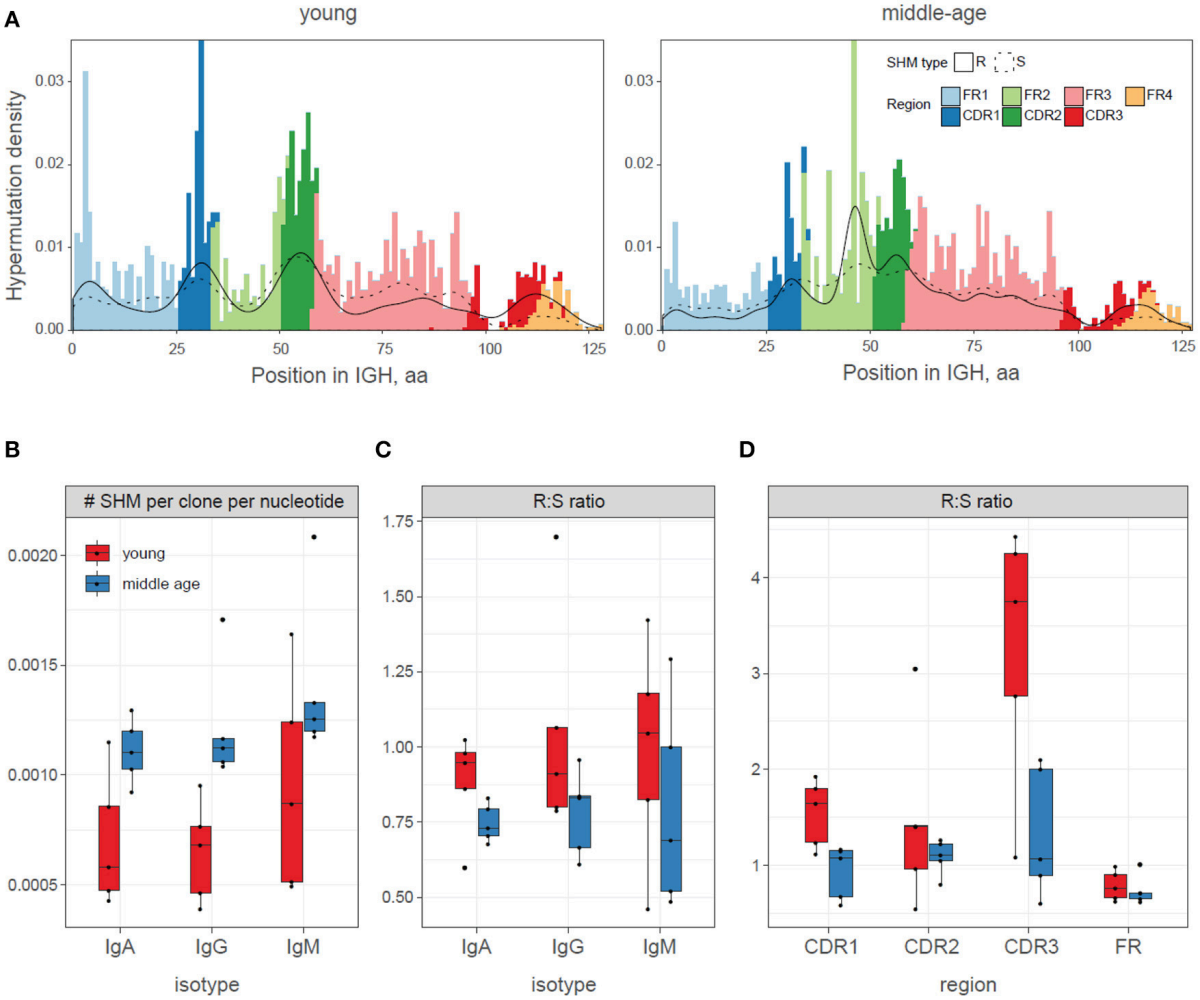
Patterns of newly acquired somatic hypermutations in young and middle-age donors vaccinated with YF. **(A)** Summary profile of somatic hypermutations observed in the study. IGH regions are marked with color. The distribution of silent (S) and replacement (R) hypermutations are shown with dashed and solid lines, respectively. For CDR3 region, mutation analysis was done using the root as reference. Data were pooled for all young and all middle-age individuals. **(B)** Frequency of newly acquired somatic hypermutations (SHMs) in young (red) and middle-age (blue) donors. ANOVA *p* = 0.0005 for age, 0.14 for isotype. **(C,D)** Mean replacement:silent ratio (R:S ratio) for newly acquired somatic hypermutations (SHMs) in young and middle-age donors, for isotypes (**C**, ANOVA *p* = 0.058 for age, 0.69 for isotype) and regions (**D**, ANOVA *p* = 0.0013 for age, 0.00014 for regions).

Finally, we focused on the newly generated mutations that are directly observed (identified in the edges of the trees), i.e., hypermutations that are supported by both observed “parent” and “child” clonotypes in the dataset. A dedicated tree building algorithm allowed us to infer the set of currently ongoing hypermutations on the entire length of the immunoglobulin sequence (Figure 5A). Note that in the full length analysis of clonal IGH evolution, we are able to identify all hypermutations that occurred within V gene segment, by comparing with the germline. For CDR3 region, we are only able to identify those mutations that differentiate the evolving clones from the identified root. We cannot determine the exact original CDR3 sequence that was generated during IGH recombination and thus cannot identify the hypermutations that have not been sampled by our analysis. This explains the lower proportion of hypermutations observed with CDR3 compared to CDR1 and CDR2 regions.

Middle-age individuals had higher total numbers of the newly acquired unique somatic hypermutations (Figure 5B), while the replacement-to-silent (R:S) ratio among such hypermutations was significantly lower compared to the young donors (Figures 5C,D).

## Discussion

Our comparative analysis of immune response to a novel pathogen, performed using immune repertoire sequencing and modeled by a live YF vaccine, revealed several differences between the two age groups indicating that humoral adaptive response already undergoes significant changes by the age of 50.

First, physicochemical properties of the hypervariable IGH CDR3 region that are linked to antigen recognition (58, 59) changed significantly (Figure 1). Differences in the interaction “energy,” Kidera factor 4, “strength” and “volume” indicate the increase of relative number of bulky, hydrophobic and strongly interacting amino acid residues in the middle of CDR3 with aging, potentially associated with increased cross-reactivity (60). In agreement with Wu et al. (43), responding repertoire of the middle-age donors also displayed longer CDR3s.

Second, the analysis of clonal lineages suggests that young individuals produce a more diverse IGH repertoire implying higher efficiency of the adaptive immune response (61). Middle-age individuals responded to YF vaccination with higher proportion of clonal hypermutating B cell trees of a larger size (Figure 3), which echoes the observation that elderly individuals have generally decreased numbers of B-cell lineages (25). Older individuals responded with lower lineage diversity, for which general decrease of B cell diversity with aging, correlating with the health status, could be one of the reasons (6, 62). While the exact reason behind the observed differences in the structure of IGH repertoire responding to novel antigens in young and middle-age individuals is unknown, one can speculate that they could be attributed to overall decrease in circulating B cells (63, 64), including memory B cells that initially recognized unrelated antigens but could respond to YF vaccination, decreased production and counts of naive B-cells (65, 66) and their diminished ability to enter somatic hypermutation (66, 67). All these factors narrow the capability of B cell immunity to select novel immunoglobulin variants.

Third, responding clonal lineages of the middle-age individuals hypermutated more intensely (Figure 5B) but less efficiently in terms of replacement-to-silent mutations ratio compared to young individuals (Figures 4C, 5C,D). The latter result overlaps with previous works, which show that loss of functional repertoire diversity is determined by not only the reducing the number of different B cell lineages but also by decreased proportion of replacement mutations (68) and mutations prominently changing the amino acid properties (32). This observation suggests that the parameters of affinity maturation may essentially vary between young and middle-age individuals, and is in line with previous works demonstrating impaired ability to produce high affinity protective antibodies against newly encountered antigens in the older individuals (13), and general changes in the mechanisms of IGH affinity maturation and memory B cells generation with aging (66).

Of note, it was earlier demonstrated that AID levels and intensity of hypermutation decrease with aging, but only after the age of 60 (63, 69, 70)—beyond the age of the cohorts that we have studied in the current work.

We have not obtained data on serum titers of YF-specific antibodies due to technical unavailability of samples. In general, it is known that the serum titers are similar for these age groups (71, 72), and are sufficiently high to provide protection for many years: (73). Thus the observed differences in the B cell response to the live YF vaccine between young and middle-age individuals are not detrimental for generation of protective immunoglobulin repertoire. However, these differences reveal the dynamics of changes in humoral response architecture that are already detectable by the age of 50 years.

Further studies involving a larger set of novel antigens and comprehensive longitudinal tracking of the response, as well as studies of vaccinations with vaccine boost, can shed more light on the fine properties of age-related changes in B cell response.

## Author contributions

DS and GS performed cell sorting. AD, AO, ML, and MS analyzed the data. AO, MS, and DC prepared the figures. ML, MT, EM, and OK worked on samples, library preparation, and sequencing. OB and DC designed and managed the entire study. MS and DC wrote the manuscript. All authors reviewed and approved the final manuscript.

### Conflict of interest statement

The authors declare that the research was conducted in the absence of any commercial or financial relationships that could be construed as a potential conflict of interest.
