# Supplementary material for: Comparative Analysis of B-Cell Receptor Repertoires Induced by Live Yellow Fever Vaccine in Young and Middle-Age Donors
Source: Front Immunol. 2018 Oct 9;9:2309. doi: 10.3389/fimmu.2018.02309 (PMC6189279; doi:10.3389/fimmu.2018.02309)
Supplement: Supplementary file 1 [file Image_1.pdf]

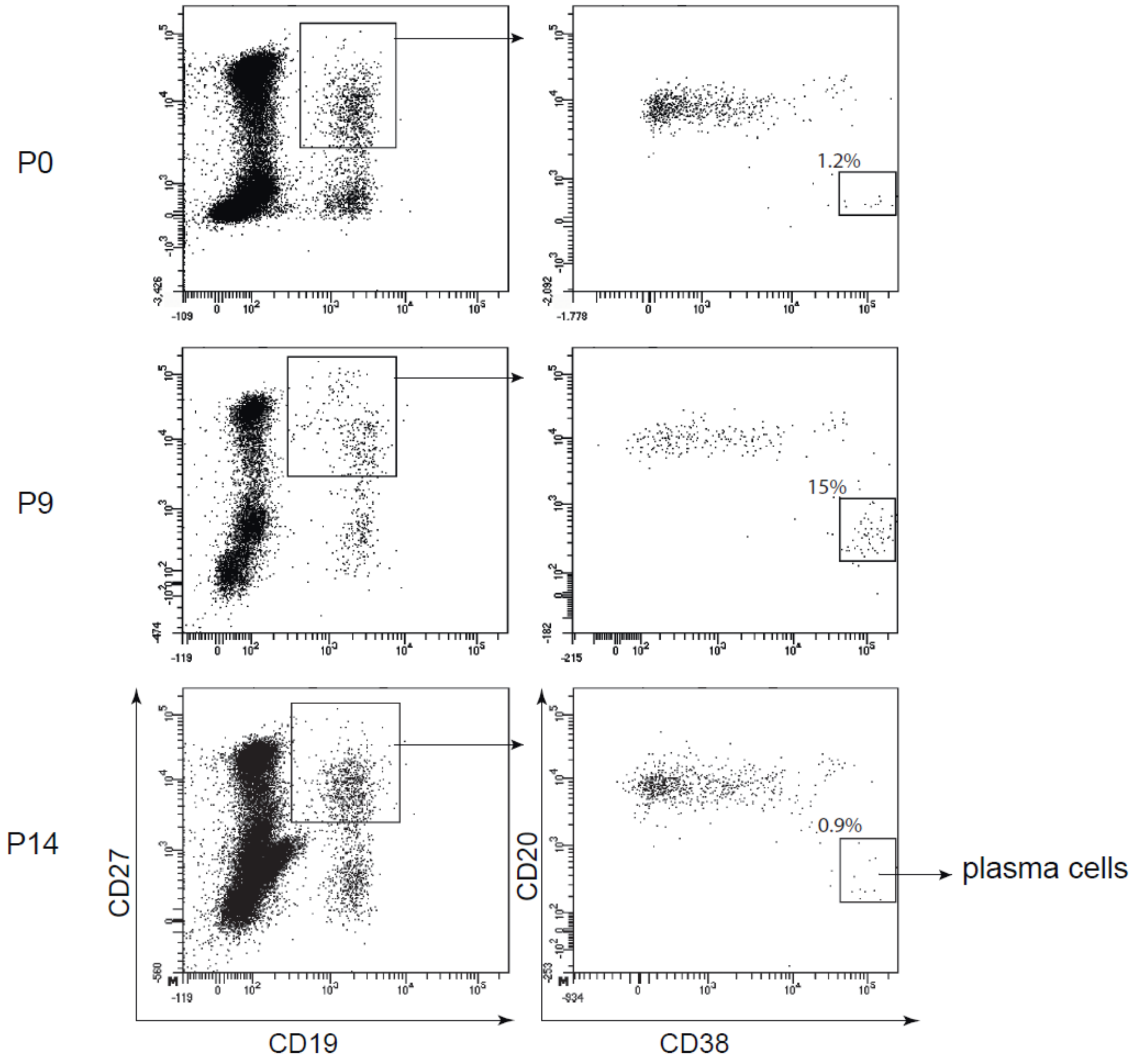

Supplementary Figure 1. Typical flow cytometry plots showing relative abundance of CD20-CD19+CD27^{high}CD38^{high} plasma B cells in peripheral blood at the day of vaccination and at time points 9 days and 14 days after vaccination.
